# Supplementary material for: Building resilient cervical cancer prevention through gender-neutral HPV vaccination
Source: eLife. 2023 Jul 24;12:e85735. doi: 10.7554/eLife.85735 (PMC10365835; doi:10.7554/eLife.85735)
Supplement: Supplementary file 1. [file elife-85735-supp1.docx]

**Supplementary file 1. List of model parameters.**

| **Notation** | **Description** | **Values / ranges** | **Reference** |
| --- | --- | --- | --- |
| *Demography* | | | |
| $m_{g, age}$ | Gender- and age-specific mortality | See **Appendix 1-Table 4** | Based UN life tables of India in 2015-2020.^1^ |
| $b$ | Population sex-specific birth rate | Set such that the total population stays constant given $m_{g, age}$ and 50%-50% distribution of female and male new-borns | NA |
| *Sexual contact behaviour* | | | |
| $p_{W,1}, p_{M,1}$ | Probability of being assigned to risk group 1 in women and men | 1.3%, 3.3% for West Bengal 1.7%, 2.8% for Tamil Nadu | Proportion of virgins in the age group 30-49 estimated by DHS in West Bengal and Tamil Nadu.^2^ |
| $p_{M,3},p_{M,4},p_{M,5}$ | Probability of being assigned to risk group 3, 4, and 5 in men | 3.6%, 4.7%, 1.4% for West Bengal 7.9%, 4.6%, 2.2% for Tamil Nadu | Based on Table 1 of Gaffey et al.^3^ The rows for low- and high-HIV states were used for West Bengal and Tamil Nadu, respectively. Risk groups 3, 4 and 5 were based on the proportion of sexually active married men having non-regular partners, sexually active unmarried men having non-regular partners but not using female sex workers, and sexually active unmarried men using female sex worker, respectively. |
| $p_{W,5}$ | Probability of being assigned to risk group 5 in women, representing female sex workers | 0.5% for West Bengal and Tamil Nadu | Estimated prevalence of female sex workers for India in Table 2 of Vandepitte et al.^4^ |
| $p_{W,3},p_{W,4}$ | Probability of being assigned to risk groups 3 and 4 in women | 2.5%, 2.5% for West Bengal  2.65%, 2.65% for Tamil Nadu | Urban percentage of women with at least one non-regular partners last year after subtracting the proportion of female sex workers in Table 6.2 of the NACO report.^5^ |
| $p_{W,2}, p_{M,2}$ | Probability of being assigned to risk group 2 in women and men | 86.0%, 93.2% for West Bengal 82.5%, 92.5% for Tamil Nadu | Remaining proportion after fixing the proportions of other risk groups. |
| $d_{W,10-14}^{stable}$, $d_{M,10-14}^{stable}$ | Population proportion with stable partnerships in women and men aged in the age group 10-14 | 0% | Set as zero given the low proportion of married individuals in this age groups as estimated by DHS.^2^ |
| $d_{W,15-19}^{stable},d_{W,20-24}^{stable},$  $d_{W,25-29}^{stable},d_{W,30-49}^{stable},$  $d_{W,50-59}^{stable},d_{W,60-99}^{stable}$ | Population proportion with stable partnerships in women in age groups 15-19, 20-24, 25-29, 30-49, 50-59, and 60-99 | 38.5%, 77.6%, 91.1%, 87.8%, 83%, 83% for West Bengal  12.8%, 58.5%, 84.7%, 86.2%, 64%, 64% for Tamil Nadu | Proportion of women married in DHS in West Bengal and Tamil Nadu.^2^ |
| $d_{M,15-19}^{stable},d_{M,20-24}^{stable},$  $d_{M,25-29}^{stable},d_{M,30-49}^{stable},$  $d_{M,50-59}^{stable},d_{M,60-99}^{stable}$ | Age-group-specific population proportion with stable partnerships in men in age groups 15-19, 20-24, 25-29, 30-49, 50-59, and 60-99 | 2.4%, 34.8%, 65.8%, 91.6%, 92.3, 92.3% for West Bengal  0.3%, 15.2%, 50.1%, 93.1%, 96.9, 96.9% for Tamil Nadu | Proportion of women married in DHS in West Bengal and Tamil Nadu.^2^ |
| $d_{W,1}^{stable}, d_{W,4}^{stable}, d_{W,5}^{stable},$  $d_{M,1}^{stable}, d_{M,4}^{stable}, d_{M,5}^{stable}$ | Population proportion with stable partnerships in risk groups 1, 4, and 5 | 0%, by definition. | NA |
| $d_{W,2}^{stable}, d_{W,3}^{stable}$  $d_{M,2}^{stable}, d_{M,3}^{stable}$ | Population proportion with stable partnerships in risk groups 2 and 3 | Set to match the age-group-specific population proportion with stable partnerships:  75% for West Bengal  72% for Tamil Nadu | NA |
| $d^{stable}$ | Overall population proportion with stable partnerships | Set to match the age-group-specific population proportion with stable partnerships:  68% for West Bengal  65% for Tamil Nadu | NA |
| $\kappa^{stable}$ | Mean age difference in stable partnerships (men minus women) | 7.29 years for West Bengal  6.58 years for Tamil Nadu | Estimated from DHS for West Bengal and Tamil Nadu.^2^ |
| $\omega^{main}$ | Mean spread of absolute age difference in stable partnerships | 3.31 years for West Bengal  3.28 years for Tamil Nadu | Estimated from DHS for West Bengal and Tamil Nadu.^2^ |
| $s^{stable}$ | Mean duration of stable partnerships | 20 years, by assumption. | NA |
| $d_{W,1}^{one-off},d_{W,2}^{one-off},$  $d_{M,1}^{one-off},d_{M,2}^{one-off}$ | Mean degree of one-off partnerships in risk groups 1 and 2 | 0%, by definition. | NA |
| $d_{M,3}^{one-off},d_{M,4}^{one-off},d_{M,5}^{one-off}$ | Mean degree of one-off partnerships in risk groups 3, 4, and 5 in men | 1.65, 1.65, 25.57 for West Bengal  3.41, 3.41, 70.58 for Tamil Nadu | Derived on in Table 6.4 of the NACO report.^5^ |
| $d_{W,3}^{one-off},d_{W,4}^{one-off}$ | Mean degree of one-off partnerships in risk groups 3 and 4 in women | 2, by assumption. | NA |
| $d_{W,5}^{one-off}$ | Sex- and age-group-specific mean degree of one-off partnerships | Set to match the degree of one-off partnerships in men:  89.1 partners per year in West Bengal  373.4 partners per year in Tamil Nadu | NA |
| $d^{one-off}$ | Overall mean degree of one-off partnerships | Set to match the risk-group-specific degree with one-off partnerships:  0.50 partners per year for West Bengal  1.98 partners per year for Tamil Nadu | NA |
| $s^{one-off}$ | Mean duration of one-off partnerships | 1 unit of time step, by definition | NA |
| $r^{stable}$ | Mean number of sex acts per time step within stable partnerships according to a Poisson distribution |  |  |
| $r^{one-off}$ | Fixed number of sex acts per time step within one-off partnerships | 1 act, by definition | NA |
| $\theta^{one-off}$ | One-off partnership underreporting rate | Derived from model calibration with the following range for candidate values: (8, 20) | NA |
| *HPV natural history* | | | |
| $\beta_{i}$ | Transmission probability of type $i$ per sex act | Derived from model calibration with the following range for candidate values: (0.2, 0.95). | NA |
| $\gamma_{16}, \gamma_{18},\gamma_{cross}, \gamma_{other}$ | Clearance rate from CIN0 | 0.824 per year for HPV 16,  0.955 per year for HPV 18,  1.18 per year for the cross-protective types and remaining HR HPV types | ^6,7^ |
| $\eta_{16}, \eta_{18},\eta_{cross}, \eta_{other}$ | Progression rate from CIN0 to CIN1 | 0.676 per year for HPV 16,  0.545 per year for HPV 18,  0.324 per year for the cross-protective types and remaining HR HPV types | ^6,7^ |
| $\delta_{16,1}, \delta_{18,1}$,$\delta_{cross,1}$, $\delta_{other,1}$ | Clearance rate from CIN1 | 0.133 per year for HPV 16,  0.386 per year for HPV 18,  0.481 per year for the cross-protective types and remaining HR HPV types | ^6,7^ |
| $\delta_{16,2}$,$\delta_{18,2},\delta_{cross,2}$,$\delta_{cross,2}$ | Clearance rate from regressive CIN2/3 | 2.10 per year for HPV 16,  2.10 per year for HPV 18,  2.10 per year for the cross-protective types and remaining HR HPV types | ^6,7^ |
| $\nu_{16,1}$,$\nu_{18,1}$,$\nu_{cross,1}$, $\nu_{other,1}$ | Progression rate from CIN1 to regressive CIN2/3 | 0.048 per year for HPV 16,  0.00681 per year for HPV 18,  0.0447 per year for the cross-protective types and remaining HR HPV types | ^6,7^ |
| $\nu_{16,2}$,$\nu_{18,2}$,$\nu_{cross,2}$, $\nu_{other,2}$ | Progression rate from CIN1 to non-regressive CIN2/3 | 0.0454 per year for HPV 16,  0.0450 per year for HPV 18,  0.0110 per year for the cross-protective types and remaining HR HPV types |  |
| $\mu_{16}$, $\mu_{18}$,$\mu_{cross}$,$\mu_{other}$ | Rate of waning natural immunity | 0.0407 per year for HPV 16,  0.0287 per year for HPV 18,  0.0320 per year for the cross-protective types and remaining HR HPV types | ^6,7^ |

# References

1. UN Life tables. United Nations Department of Economic Social Affairs Population Dynamics.
2. The Demographic and Health Surveys (DHS). United States Agency of International Development (USAID).
3. Gaffey MF, Venkatesh S, Dhingra N, et al. Male use of female sex work in India: A nationally representative behavioural survey. *PLoS One* 2011; **6**(7): e22704.
4. Vandepitte J, Lyerla R, Dallabetta G, Crabbé F, Alary M, Buvé A. Estimates of the number of female sex workers in different regions of the world. *Sexually Transmitted Infections* 2006; **82**(suppl 3): iii18-25.
5. National Behavioural Surveillance Survey: General Population 2006. National AIDS Control Organisation Ministry of Health and Family Welfare Government of India.
6. Berkhof J, Bogaards JA, Demirel E, Diaz M, Sharma M, Kim JJ. Cost-Effectiveness of Cervical Cancer Prevention in Central and Eastern Europe and Central Asia. *Vaccine* 2013; **31**(S7): H71-H9.
7. Bogaards JA, Xiridou M, Coupé VMH, Meijer CJLM, Wallinga J, Berkhof J. Model-based estimation of viral transmissibility and infection-induced resistance from the age-dependent prevalence of infection for 14 high-risk types of human papillomavirus. *American Journal of Epidemiology* 2010; **171**(7): 817-25.
