# Supplementary material for: Building resilient cervical cancer prevention through gender-neutral HPV vaccination
Source: eLife. 2023 Jul 24;12:e85735. doi: 10.7554/eLife.85735 (PMC10365835; doi:10.7554/eLife.85735)
Supplement: Supplementary file 2. — (A) Overview of available cancer incidence data from local registries by Indian state. (B) Age-specific cervical cancer incidence data by Indian state. (C) Mortality rate of India. (D) Type-specific contribution of HPV types in cervical cancer. (E) Standard world population (Segi, 1960). (F) Female population size by Indian state.(G) Pre-vaccination risk of cervical cancer by Indian state. [file elife-85735-supp2.docx]

**Supplementary file 2A. Overview of available cancer incidence data from local registries by Indian state.*** States or groups of states as reported in the 2006 National Behaviour Surveillance Survey of the National AIDS Control Organization of India.^1^
§ Other North Eastern States include Arunachal Pradesh, Nagaland, Meghalaya, Mizoram, and Tripura.
¤ The eighteen registries CI5 and NCDIR do not have in common are in *italics*.

| **State/group of states *** | **CI5 registry** ¤ ^2^ | **NCDIR registry** ¤ ^3^ |
| --- | --- | --- |
| Andhra Pradesh |  | *Hyderabad district* |
| Assam | Cachar, Kamrup Urban District | Cachar district, *Dibrugarh district*, Kamrup urban |
| Bihar |  |  |
| Chhattisgarh |  |  |
| Delhi |  | *Delhi* |
| Goa + Daman & Diu |  |  |
| Gujarat + Dadra & Nagar Haveli | Ahmedabad | Ahmedabad urban |
| Haryana |  |  |
| Himachal Pradesh |  |  |
| Jammu & Kashmir |  |  |
| Jharkhand |  |  |
| Karnataka | Bangalore | Bangalore |
| Kerala + Lakshadweep | Kollam, Trivandrum | Kollam district, Thi'puram district |
| Madhya Pradesh | Bhopal | Bhopal |
| Maharashtra | Barshi & Paranda & Bhum, Mumbai, Poona, Wardha | *Aurangabad*, *Osamanabad & Beed*, Barshi rural, Mumbai, Pune, Wardha district, *Nagpur* |
| Manipur |  | *Manipur state*, *Imphal West district* |
| Orissa |  |  |
| Other North Eastern States § | Mizoram, Tripura | Mizoram state, *Aizawl district*, Tripura state, *West Arunachal, Papumpare district, Meghalaya, East Khasi Hills district, Nagaland, Pasighat* |
| Punjab + Chandigarh |  | *Patiala district* |
| Rajasthan |  |  |
| Sikkim | Sikkim State | Sikkim state |
| Tamil Nadu + Puducherry | Chennai, *Dindigul Ambilikkai* | Chennai |
| Uttar Pradesh |  |  |
| Uttarakhand |  |  |
| West Bengal + Andaman & Nicobar Islands |  | *Kolkata* |

**Supplementary file 2B. Age-specific cervical cancer incidence data by Indian state.**Incidence is given in cases per 100,000 woman-years by 5-year age groups.
* States or groups of states as reported in the 2006 National Behaviour Surveillance Survey of the National AIDS Control Organization of India.^1^

§ Other North Eastern States include Arunachal Pradesh, Nagaland, Meghalaya, Mizoram, and Tripura.
₮ “Extracted”: cervical cancer incidence data were extracted from CI5 or NCDIR when available; “Inferred”: when cervical cancer incidence data were unavailable, they were inferred based on footprinting.^2–4^
¤ Belonging to low- or high-incidence cluster. Cluster was obtained by the clustering step when cervical cancer incidence data were available and by the classification step whenever cervical cancer incidence data were unavailable.

|  |  |  |  | **Age group** | | | | | | | | | | | | | |
| --- | --- | --- | --- | --- | --- | --- | --- | --- | --- | --- | --- | --- | --- | --- | --- | --- | --- |
| **State/group of states *** | **Source** ₮ |  | **Cluster** ¤ | **15-19** | **20-24** | **25-29** | **30-34** | **35-39** | **40-44** | **45-49** | **50-54** | **55-59** | **60-64** | **65-69** | **70-74** | **75-79** | **80-84** |
| Andhra Pradesh | Extracted |  | Low | 0 | 0.1 | 1.5 | 3.6 | 11.2 | 15.7 | 20.3 | 35.3 | 44.6 | 51 | 43.8 | 55.7 | 25.7 | 12.8 |
| Assam | Extracted |  | Low | 0 | 0.2 | 1.5 | 5.1 | 7.9 | 15.1 | 24.1 | 27.9 | 28.2 | 33.2 | 36.1 | 25.5 | 13.9 | 7 |
| Bihar | Inferred |  | Low | 0 | 0.3 | 1 | 4.3 | 8.4 | 16.2 | 22.2 | 28.6 | 30.7 | 34.9 | 31.9 | 31.9 | 15.3 | 7.6 |
| Chhattisgarh | Inferred |  | Low | 0 | 0.3 | 1 | 4.3 | 8.4 | 16.2 | 22.2 | 28.6 | 30.7 | 34.9 | 31.9 | 31.9 | 15.3 | 7.6 |
| Delhi | Extracted |  | High | 0 | 0.6 | 1.2 | 4.4 | 11.2 | 21.8 | 32.3 | 38.5 | 45.4 | 62.3 | 57 | 51.4 | 31.9 | 16 |
| Goa + Daman & Diu | Inferred |  | Low | 0 | 0.3 | 1 | 4.3 | 8.4 | 16.2 | 22.2 | 28.6 | 30.7 | 34.9 | 31.9 | 31.9 | 15.3 | 7.6 |
| Gujarat + Dadra & Nagar Haveli | Extracted |  | Low | 0 | 0 | 0.8 | 7.9 | 14 | 24.2 | 19.7 | 34.4 | 19.6 | 30.6 | 25.7 | 30.6 | 13.6 | 6.8 |
| Haryana | Inferred |  | Low | 0 | 0.3 | 1 | 4.3 | 8.4 | 16.2 | 22.2 | 28.6 | 30.7 | 34.9 | 31.9 | 31.9 | 15.3 | 7.6 |
| Himachal Pradesh | Inferred |  | Low | 0 | 0.3 | 1 | 4.3 | 8.4 | 16.2 | 22.2 | 28.6 | 30.7 | 34.9 | 31.9 | 31.9 | 15.3 | 7.6 |
| Jammu & Kashmir | Inferred |  | Low | 0 | 0.3 | 1 | 4.3 | 8.4 | 16.2 | 22.2 | 28.6 | 30.7 | 34.9 | 31.9 | 31.9 | 15.3 | 7.6 |
| Jharkhand | Inferred |  | Low | 0 | 0.3 | 1 | 4.3 | 8.4 | 16.2 | 22.2 | 28.6 | 30.7 | 34.9 | 31.9 | 31.9 | 15.3 | 7.6 |
| Karnataka | Extracted |  | High | 0 | 0.2 | 0.9 | 4.9 | 10 | 23.8 | 40.4 | 52.2 | 63.7 | 64 | 79.6 | 74.1 | 30.2 | 15.1 |
| Kerala + Lakshadweep | Extracted |  | Low | 0 | 0.2 | 0.4 | 0.3 | 2.5 | 9.2 | 15 | 19.1 | 30 | 38.4 | 31.9 | 39.3 | 11.7 | 5.8 |
| Madhya Pradesh | Extracted |  | High | 0.2 | 0.2 | 2 | 4.3 | 12.8 | 23.8 | 30.4 | 53.3 | 48.8 | 65 | 61.8 | 76.4 | 17.7 | 8.8 |
| Maharashtra | Extracted |  | Low | 0 | 0.4 | 1.3 | 3.8 | 10.8 | 20.8 | 27.1 | 35.4 | 36.8 | 50.5 | 51.7 | 43.3 | 22.4 | 11.2 |
| Manipur | Extracted |  | Low | 0 | 0.6 | 0.1 | 4.2 | 7.4 | 12.2 | 14.8 | 21.3 | 25.1 | 35 | 32.7 | 39.4 | 23.2 | 11.6 |
| Orissa | Inferred |  | Low | 0 | 0.3 | 1 | 4.3 | 8.4 | 16.2 | 22.2 | 28.6 | 30.7 | 34.9 | 31.9 | 31.9 | 15.3 | 7.6 |
| Other North Eastern States § | Extracted |  | High | 0 | 1.1 | 1.9 | 10.7 | 20.3 | 40.9 | 50 | 54.3 | 52.2 | 47.8 | 31.9 | 44 | 16.6 | 8.3 |
| Punjab + Chandigarh | Extracted |  | High | 0 | 0.1 | 2.2 | 3 | 13 | 23.8 | 32.3 | 47.2 | 47.8 | 48.7 | 52.8 | 35.2 | 33.3 | 16.6 |
| Rajasthan | Inferred |  | Low | 0 | 0.3 | 1 | 4.3 | 8.4 | 16.2 | 22.2 | 28.6 | 30.7 | 34.9 | 31.9 | 31.9 | 15.3 | 7.6 |
| Sikkim | Extracted |  | Low | 0 | 0 | 0.7 | 4.3 | 12 | 26.1 | 29 | 24.6 | 42.8 | 26.5 | 20.8 | 12.4 | 0 | 0 |
| Tamil Nadu + Puducherry | Extracted |  | High | 0 | 0.4 | 0.9 | 4.3 | 13.4 | 33.7 | 48.1 | 60.8 | 68.9 | 75.5 | 67.1 | 65.1 | 19.9 | 9.9 |
| Uttar Pradesh | Inferred |  | Low | 0 | 0.3 | 1 | 4.3 | 8.4 | 16.2 | 22.2 | 28.6 | 30.7 | 34.9 | 31.9 | 31.9 | 15.3 | 7.6 |
| Uttarakhand | Inferred |  | Low | 0 | 0.3 | 1 | 4.3 | 8.4 | 16.2 | 22.2 | 28.6 | 30.7 | 34.9 | 31.9 | 31.9 | 15.3 | 7.6 |
| West Bengal + Andaman & Nicobar Islands | Extracted |  | Low | 0 | 0.3 | 0.6 | 3.5 | 6.2 | 15.9 | 23.7 | 27.5 | 26.7 | 29.1 | 34.8 | 31.7 | 21.5 | 10.8 |

**Supplementary file 2C. Mortality rate of India.**Obtained from UN life tables for 2015-2020.^5^

| **Age group** | **Women** | **Men** |
| --- | --- | --- |
| 0-0 | 0.03290 | 0.05072 |
| 1-4 | 0.00218 | 0.00339 |
| 5-9 | 0.00075 | 0.00116 |
| 10-14 | 0.00061 | 0.00087 |
| 15-19 | 0.00100 | 0.00129 |
| 20-24 | 0.00129 | 0.00189 |
| 25-29 | 0.00136 | 0.00231 |
| 30-34 | 0.00154 | 0.00296 |
| 35-39 | 0.00201 | 0.00401 |
| 40-44 | 0.00290 | 0.00522 |
| 45-49 | 0.00400 | 0.00744 |
| 50-54 | 0.00761 | 0.01092 |
| 55-59 | 0.01060 | 0.01633 |
| 60-64 | 0.01770 | 0.02493 |
| 65-69 | 0.02730 | 0.03843 |
| 70-74 | 0.04430 | 0.05652 |
| 75-79 | 0.06650 | 0.08317 |
| 80-84 | 0.10800 | 0.12841 |
| 85-89 | 0.16400 | 0.19412 |
| 90-94 | 0.24200 | 0.28397 |
| 95-99 | 0.22500 | 0.26828 |

**Supplementary file 2D. Type-specific contribution of HPV types in cervical cancer.**Obtained from a study of HPV distribution in cervical cancer in India.^6^

| **HPV type** | **16** | **18** | **31** | **33** | **35** | **39** | **45** | **52** | **56** | **58** | **59** | **68** | **73** | **other** | **Contribution of a given combination of HPV types (%)** |
| --- | --- | --- | --- | --- | --- | --- | --- | --- | --- | --- | --- | --- | --- | --- | --- |
| **Combination of HPV types** | x |  |  |  |  |  |  |  |  |  |  |  |  |  | 57.5 |
|  |  | x |  |  |  |  |  |  |  |  |  |  |  |  | 10.4 |
|  |  |  | x |  |  |  |  |  |  |  |  |  |  |  | 1.0 |
|  |  |  |  | x |  |  |  |  |  |  |  |  |  |  | 3.1 |
|  |  |  |  |  |  | x |  |  |  |  |  |  |  |  | 0.5 |
|  |  |  |  |  |  |  | x |  |  |  |  |  |  |  | 1.6 |
|  |  |  |  |  |  |  |  | x |  |  |  |  |  |  | 1.0 |
|  |  |  |  |  |  |  |  |  | x |  |  |  |  |  | 1.6 |
|  |  |  |  |  |  |  |  |  |  | x |  |  |  |  | 1.6 |
|  |  |  |  |  |  |  |  |  |  |  | x |  |  |  | 2.1 |
|  | x | x |  |  |  |  |  |  |  |  |  |  |  |  | 7.3 |
|  | x |  | x |  |  |  |  |  |  |  |  |  |  |  | 0.5 |
|  | x |  |  | x |  |  |  |  |  |  |  |  |  |  | 1.0 |
|  | x |  |  |  | x |  |  |  |  |  |  |  |  |  | 1.0 |
|  | x |  |  |  |  | x |  |  |  |  |  |  |  |  | 0.5 |
|  | x |  |  |  |  |  |  | x |  |  |  |  |  |  | 1.0 |
|  | x |  |  |  |  |  |  |  | x |  |  |  |  |  | 0.5 |
|  | x |  |  |  |  |  |  |  |  | x |  |  |  |  | 0.5 |
|  | x |  |  |  |  |  |  |  |  |  |  | x |  |  | 0.5 |
|  |  |  |  |  |  |  |  |  |  |  |  |  | x |  | 0.5 |
|  |  | x |  |  | x |  |  |  |  |  |  |  |  |  | 0.5 |
|  |  |  |  |  |  |  |  |  |  |  |  |  |  | x | 5.8 |
| **Unnormalised contributions (%)** | 70 | 18 | 2 | 4 | 2 | 1 | 2 | 2 | 2 | 2 | 2 | 1 | 1 | 6 |  |
| **Normalised contributions (%)** | 62 | 16 | 1 | 4 | 1 | 1 | 1 | 2 | 2 | 2 | 2 | 0 | 0 | 5 |  |

**Supplementary file 2E. Standard world population.^7^**

| **Age group** | | **Population** |  |
| --- | --- | --- | --- |
| 0-4 | 12000 | |  |
| 5-9 | 10000 | |  |
| 10-14 | 9000 | |  |
| 15-19 | 9000 | |  |
| 20-24 | 8000 | |  |
| 25-29 | 8000 | |  |
| 30-34 | 6000 | |  |
| 35-39 | 6000 | |  |
| 40-44 | 6000 | |  |
| 45-49 | 6000 | |  |
| 50-54 | 5000 | |  |
| 55-59 | 4000 | |  |
| 60-64 | 4000 | |  |
| 65-69 | 3000 | |  |
| 70-74 | 2000 | |  |
| 75-79 | 1000 | |  |
| 80-84 | 500 | |  |
| 85+ | 500 | |  |
| Total | 100000 | |  |

**Supplementary file 2F. Female population size by Indian state.**Extracted from table C-13 by the Indian Census.^8^
* States or groups of states as reported in the 2006 National Behaviour Surveillance Survey of the National AIDS Control Organization of India.1
ǂ Clustering of states into groups of high and low cervical cancer incidence was derived in a separate manuscript.^4^
§ Other North Eastern States include Arunachal Pradesh, Nagaland, Meghalaya, Mizoram, and Tripura.

| **State/group of states *** | **Cluster ǂ** | **Population size** | **Percentage (%)** |
| --- | --- | --- | --- |
| Andhra Pradesh | Low | 41754886 | 7.13 |
| Assam | Low | 15257203 | 2.61 |
| Bihar | Low | 49638102 | 8.48 |
| Chhattisgarh | Low | 12701295 | 2.17 |
| Delhi | High | 7793088 | 1.33 |
| Goa + Daman & Diu | Low | 811247 | 0.14 |
| Gujarat + Dadra & Nagar Haveli | Low | 28984911 | 4.95 |
| Haryana | Low | 11842082 | 2.02 |
| Himachal Pradesh | Low | 3377919 | 0.58 |
| Jammu & Kashmir | Low | 5895268 | 1.01 |
| Jharkhand | Low | 16003337 | 2.73 |
| Karnataka | High | 30108199 | 5.14 |
| Kerala + Lakshadweep | Low | 17392769 | 2.97 |
| Madhya Pradesh | High | 34975017 | 5.97 |
| Maharashtra | Low | 53942893 | 9.21 |
| Manipur | Low | 1413663 | 0.24 |
| Orissa | Low | 20704258 | 3.54 |
| Other North Eastern States § | High | 5435403 | 0.93 |
| Punjab + Chandigarh | High | 13559265 | 2.32 |
| Rajasthan | Low | 32865353 | 5.61 |
| Sikkim | Low | 286968 | 0.05 |
| Tamil Nadu + Puducherry | High | 36611821 | 6.25 |
| Uttar Pradesh | Low | 94575702 | 16.15 |
| Uttarakhand | Low | 4941223 | 0.84 |
| West Bengal + Andaman & Nicobar Islands | Low | 44595926 | 7.62 |
| Total (Low-incidence cluster) |  | 456985005 | 78.05 |
| Total (High-incidence cluster) |  | 128482793 | 21.94 |
| Total (all states) |  | 585467798 | 100.00 |

**Supplementary file 2G. Pre-vaccination risk of cervical cancer by Indian state.*** States or groups of states as reported in the 2006 National Behaviour Surveillance Survey of the National AIDS Control Organization of India.^1^
§ Other North Eastern States include Arunachal Pradesh, Nagaland, Meghalaya, Mizoram, and Tripura.
ƚ Cases per 100,000 girls born
ǂ Cases per 100,000 woman-years

| **State/group of states *** | **Life-time risk** ƚ | **Age-standardised incidence rate** ǂ |
| --- | --- | --- |
| Andhra Pradesh | 1157 | 11.5 |
| Assam | 849 | 8.9 |
| Bihar | 870 | 9 |
| Chhattisgarh | 870 | 9 |
| Delhi | 1353 | 13.7 |
| Goa + Daman & Diu | 870 | 9 |
| Gujarat + Dadra & Nagar Haveli | 868 | 9.3 |
| Haryana | 870 | 9 |
| Himachal Pradesh | 870 | 9 |
| Jammu & Kashmir | 870 | 9 |
| Jharkhand | 870 | 9 |
| Karnataka | 1667 | 16.8 |
| Kerala + Lakshadweep | 738 | 7.3 |
| Madhya Pradesh | 1498 | 15.3 |
| Maharashtra | 1158 | 11.9 |
| Manipur | 804 | 7.9 |
| Orissa | 870 | 9 |
| Other North Eastern States § | 1483 | 16.3 |
| Punjab + Chandigarh | 1304 | 13.4 |
| Rajasthan | 870 | 9 |
| Sikkim | 820 | 9.2 |
| Tamil Nadu + Puducherry | 1764 | 18.5 |
| Uttar Pradesh | 870 | 9 |
| Uttarakhand | 870 | 9 |
| West Bengal + Andaman & Nicobar Islands | 843 | 8.6 |
| Total (Low-incidence cluster) | 922 | 9.5 |
| Total (High-incidence cluster) | 1583 | 16.3 |
| Total (all states) | 1067 | 11 |

# References

1. National Behavioural Surveillance Survey: General Population 2006. National AIDS Control Organisation Ministry of Health and Family Welfare Government of India.
2. Bray F, Colombet M, Mery L, et al. Cancer Incidence in Five Continents, Vol. XI (electronic version). Lyon: International Agency for Research on Cancer. 2017.
3. Report of National Cancer Registry Programme 2012-2016. National Centre for Disease Informatics and Research. 2020.
4. Man I, Georges D, Bonjour M, Baussano I. Approximating missing epidemiological data for cervical cancer through Footprinting: A case study. *eLife* 2023; **12**: e81752.
5. UN Life tables. United Nations Department of Economic Social Affairs Population Dynamics.
6. Basu P, Chandna P, Bamezai RNK, et al. MassARRAY spectrometry is more sensitive than PreTect HPV-Proofer and consensus PCR for type-specific detection of high-risk oncogenic human papillomavirus genotypes in cervical cancer. *Journal of Clinical Microbiology* 2011; **49**(10): 3537-44.
7. Segi M. Cancer mortality for selected sites in 24 countries 1950-57. *Sendai, Japan: Department of Public Health, Tohoku University of Medicine* 1960.
8. India Census C-series 2011. Office of the Registrar, General Census Commissioner India.
