# Supplementary material for: Building resilient cervical cancer prevention through gender-neutral HPV vaccination
Source: eLife. 2023 Jul 24;12:e85735. doi: 10.7554/eLife.85735 (PMC10365835; doi:10.7554/eLife.85735)
Supplement: Supplementary file 3. [file elife-85735-supp3.docx]

| *I. Life-time number of cervical cancer cases prevented prior to disruption* | | | | | |  |  |  |
| --- | --- | --- | --- | --- | --- | --- | --- | --- |
| Scenario | States | GO 60% | GO 90% | GN 60% | GN 90% |  |  |  |
| No disruption | All | 562 (444, 676) | 773 (701, 836) | 647 (539, 746) | 807 (752, 853) |  |  |  |
|  | High | 852 (706, 995) | 1149 (1062, 1223) | 936 (777, 1081) | 1197 (1130, 1260) |  |  |  |
|  | Low | 481 (370, 586) | 668 (599, 727) | 566 (472, 651) | 697 (646, 739) |  |  |  |
|  | | | | | | |  |  |
| *II. Sensitivity analyses on coverage at disruption (with duration of disruption fixed at 5 years)* | | | | | | |  |  |
| Coverage at disruption in % | States | Resilience by vaccination strategy and coverage | | | | Resilience ratio | | |
|  |  | GO 60% | GO 90% | GN 60% | GN 90% | GO 60% to GO 90% | GO 60% to GN 60% | GO 90% to GN 90% |
| 0 (base case) | All | 107 (7, 214) | 209 (81, 340) | 302 (170, 437) | 464 (328, 602) | 2.0 | 2.8 | 2.2 |
|  | High | 151 (33, 281) | 291 (136, 457) | 459 (291, 628) | 693 (515, 876) | 1.9 | 3.0 | 2.4 |
|  | Low | 95 (0, 195) | 186 (66, 307) | 257 (136, 383) | 399 (276, 525) | 2.0 | 2.7 | 2.2 |
| 20 | All | 271 (155, 391) | 355 (221, 490) | 425 (297, 559) | 550 (416, 680) | 1.3 | 1.6 | 1.6 |
|  | High | 393 (235, 555) | 508 (341, 678) | 638 (460, 823) | 820 (647, 989) | 1.3 | 1.6 | 1.6 |
|  | Low | 237 (132, 345) | 312 (187, 438) | 366 (251, 485) | 474 (351, 593) | 1.3 | 1.5 | 1.5 |
| 40 | All | 410 (277, 534) | 476 (343, 599) | 527 (401, 647) | 621 (500, 730) | 1.2 | 1.3 | 1.3 |
|  | High | 603 (442, 757) | 697 (533, 853) | 788 (631, 929) | 926 (777, 1060) | 1.2 | 1.3 | 1.3 |
|  | Low | 356 (231, 471) | 414 (289, 527) | 453 (337, 567) | 536 (423, 638) | 1.2 | 1.3 | 1.3 |
| *III. Sensitivity analyses on duration of disruption (with coverage at disruption fixed at 0%)* | | | | | | |  |  |
| Duration of disruption in years | States | Resilience by vaccination strategy and coverage | | | | Resilience ratio | | |
|  |  | GO 60% | GO 90% | GN 60% | GN 90% | GO 60% to GO 90% | GO 60% to GN 60% | GO 90% to GN 90% |
| 1 | All | 137 (26, 253) | 261 (125, 407) | 365 (215, 502) | 517 (372, 655) | 1.9 | 2.7 | 2.0 |
|  | High | 202 (63, 345) | 372 (183, 575) | 549 (350, 726) | 767 (605, 930) | 1.8 | 2.7 | 2.1 |
|  | Low | 119 (16, 227) | 230 (109, 359) | 313 (177, 439) | 447 (307, 578) | 1.9 | 2.6 | 1.9 |
| 2 | All | 125 (17, 233) | 240 (105, 375) | 344 (206, 480) | 500 (359, 642) | 1.9 | 2.7 | 2.1 |
|  | High | 183 (54, 324) | 338 (167, 519) | 507 (333, 692) | 738 (553, 929) | 1.9 | 2.8 | 2.2 |
|  | Low | 109 (6, 207) | 212 (87, 334) | 298 (171, 421) | 433 (305, 561) | 2.0 | 2.7 | 2.0 |
| 5 (base case) | All | 107 (7, 214) | 209 (81, 340) | 302 (170, 437) | 464 (328, 602) | 2.0 | 2.8 | 2.2 |
|  | High | 151 (33, 281) | 291 (136, 457) | 459 (291, 628) | 693 (515, 876) | 1.9 | 3.0 | 2.4 |
|  | Low | 95 (0, 195) | 186 (66, 307) | 257 (136, 383) | 399 (276, 525) | 2.0 | 2.7 | 2.2 |
| 10 | All | 80 (0, 182) | 154 (33, 275) | 226 (96, 358) | 382 (240, 525) | 1.9 | 2.8 | 2.5 |
|  | High | 113 (0, 241) | 217 (64, 367) | 340 (168, 506) | 560 (381, 741) | 1.9 | 3.0 | 2.6 |
|  | Low | 71 (0, 165) | 137 (25, 249) | 194 (76, 317) | 332 (200, 464) | 1.9 | 2.8 | 2.4 |

**Supplementary file 3. Sensitivity analyses on coverage at disruption and duration of disruption on resilience by Indian state*.*** Life-time number of cervical cancer cases prevented per 100,000 girls born in birth cohorts vaccinated prior to disruption in part I. Sensitivity analyses on coverage at disruption in part II and on duration of disruption in part III on resilience (defined as the life-time number of cervical cancer cases still prevented in the birth cohorts with disruption of vaccination per 100,000 girls born) and resilience ratio (defined as fold change in resilience by switching from one scenario to another). Uncertainty intervals are reported in brackets. [Resilience ratio of scenario X to scenario Y] is defined is [resilience of scenario Y] / [resilience of scenario X]. For example, in the base case, [resilience ratio of GO 60% to GO 90%] = [resilience of GO 90%] / [resilience of GO 60%] = 209 / 107 = 2.0.
