# Supplementary material for: Building resilient cervical cancer prevention through gender-neutral HPV vaccination
Source: eLife. 2023 Jul 24;12:e85735. doi: 10.7554/eLife.85735 (PMC10365835; doi:10.7554/eLife.85735)
Supplement: Reporting standard 1. [file elife-85735-repstand1.docx]

**Reporting standard 1. HPV-FRAME checklist** (Canfell K, Kim JJ, Kulasingam S, et al. HPV-FRAME: A consensus statement and quality framework for modelled evaluations of HPV-related cancer control. *Papillomavirus Research* 2019; **8**: 100184.)

Y=yes; N=no; F=female; M=male; NA=not applicable

| **Core reporting standard** |  |  |  |
| --- | --- | --- | --- |
| **a) Inputs** | **Reported by age? (Y/N)** | **Report by sex?  (F-only, M-only or both)** | **Comments** |
| Target population for intervention | Y | Both | Routine vaccination in girls and boys were considered. Age of routine vaccination was reported. |
| Sexual behaviour | Y | Both | Parameters reports: proportion of risk-group; age- and risk-group specific numbers of stable and one-off partnerships; rates of partnership dissolution; number of sex acts per unit of time given established partnership; probability of HPV transmission per sex act. Parameters were reported separately by model for the high and low cervical cancer incidence cluster. Derivation of assortative parameters was reported. |
| Cohort examined for evaluation / time horizon | N | F-only | Life-time risk of cervical cancer was reported for the first 40 vaccinated cohorts in the routine programme. Age-standardised incidence rate of cervical cancer was reported up to 100 years after the introduction of vaccination. |
| Quality of life assumptions | NA | NA | NA |
| Calibration | Y | Y | The standard STERGM calibration function was used to obtain age- and risk-groups parameters of partnership formation and dissolution. Subsequently, maximum likelihood was used to assess the goodness-of-fit to age-specific HPV prevalence data and to obtain HPV transmission probabilities and one-off partnership underreporting rate. |
| Validation (where possible) | Y | Y | Validation adequate cervical cancer incidence resulting from the HPV infection incidence here was reported in the accompanying paper of a previous publication.^24^ |
| Costs | NA | NA | NA |

(Table continues the next page)

| **Reporting standard for HPV vaccination in adolescent individuals** | | | | |
| --- | --- | --- | --- | --- |
| **a) Inputs** | **Reported?  (Y/N)** | **Reported by age? (Y/N)** | **Report by sex?  (F-only, M-only or both)** | **Report as calibration or validation target? (Y/N)** |
| Vaccine uptake | Y | Y | Y | Vaccination in girls and boys were considered. Uptake between 0-100% were considered. |
| Vaccine efficacy | Y | Y | NA | Efficacy by HPV type was considered. Efficacy was independent of age or gender. |
| Vaccine cross-protection | Y | Y | NA | Level of cross-protection for HPV 31/33/45 was reported. |
| Duration vaccine protection and waning | Y | Y | NA | Lifelong vaccine protection was considered based on the evidence from the IARC India vaccine trial.^25^ |
| Vaccine and delivery costs | NA | NA | NA | Economic assessment was not performed. |
| Pre-vaccination disease burden (including population attributable fractions for HPV) | Y, for cervical cancer. | Y, for cervical cancer. | F-only, for cervical cancer. | Attributable fraction by HPV type to cervical cancer burden was reported. |
| Duration of natural immunity | Y | NA | Y | Natural immunity was independent of age. Sex-specific natural immunity assumptions were reported. |
| **b) Outputs** | **Reported?  (Y/N)** | **Reported by age? (Y/N)** | **Report by sex? (F-only, M-only or both)** | **Comments** |
| Absolute reductions in HPV infections, and/or warts, post-vaccination | N | NA | NA | Impact on HPV prevalence and warts were not considered. |
| Absolute reductions in CIN2+ post-vaccination | N | NA | NA | NA |
| Absolute reductions in invasive cancer (cervical and other HPV cancers, as relevant) | Y, for cervical cancer. | N | F-only, cervical cancer. | NA |

| **Reporting standard for models of HPV prevention in LMIC** | | |  |  |
| --- | --- | --- | --- | --- |
| **a) Inputs** | **Reported? (Y/N)** | **Reported by age? (Y/N)** | **Report by sex?  (F-only, M-only or both)** | **Comments** |
| HIV prevalence rates if endemic in country | N | N | N | The effects of HIV are not modelled because of the low HIV prevalence in India.^6^ |
| Description of any opportunistic or pilot/demonstration screening project ongoing | Y | NA | NA | Opportunistic cervical cancer screening is done in the Indian national communicable disease control programme. However, the coverage is very low. Ever-in-lifetime coverage was reported to be lower than 3% for women aged 30–49 years in the 2017–18 NCD monitoring survey.^26^ Hence, no screening was assumed in the present study. |

| **Reporting standards for evaluations assessing alternative vaccine types or reduced-dose schedules** | | | | |
| --- | --- | --- | --- | --- |
| **a) Inputs** | **Reported? (Y/N)** | **Reported by age? (Y/N)** | **Report by sex?  (F-only, M-only or both)** | **Comments** |
| Vaccine efficacy/waning | Y | See Comments. | See Comments. | Efficacy by HPV type was considered. Efficacy was independent of age or gender. Lifelong vaccine protection was considered based on the evidence from the IARC India vaccine trial representing single-, two-, or three-dose scehdules.^25^ |
| Timing between doses (for 2-dose) | NA | NA | NA | Timing between the two doses under two-dose vaccination schedule was not modelled. We assumed constant and life-long efficacy. |
| Vaccine cross-protection | Y | See Comments. | See Comments. | Level of cross-protection for HPV 31/33/45 was reported. Cross-protection was independent of age or gender. |
| Cost | NA | NA | NA | NA |
| **b) Outputs** | **Reported? (Y/N)** | **Reported by age? (Y/N)** | **Report by sex? (F-only, M-only or both)** | **Report as calibration or validation target (Y/N)?** |
| Threshold cost per dose | NA | NA | NA | NA |

# 
